# Radiation Sciences Education in Africa: An Assessment of Current Training Practices and Evaluation of a High-Yield Course in Radiation Biology and Radiation Physics

**DOI:** 10.1200/GO.20.00350

**Published:** 2020-10-27

**Authors:** Shane R. Stecklein, Cullen M. Taniguchi, Adam D. Melancon, Dorothy Lombe, Kennedy Lishimpi, Lewis Banda, Catherine Mwaba, George Pupwe, Maurice Mwale, Harry Munkupa, Mulape Kanduza, Barbara Mule, Augustine Mwale, Laurence Court, Jared D. Ohrt, Michael E. Kupferman, Anuja Jhingran, Susan Citonje Msadabwe-Chikuni

**Affiliations:** ^1^ Department of Radiation Oncology, The University of Kansas School of Medicine, Kansas City, KS; ^2^ The University of Kansas Cancer Center, Kansas City, KS; ^3^ Department of Radiation Oncology, The University of Texas MD Anderson Cancer Center, Houston, TX; ^4^ Department of Radiation Physics, The University of Texas MD Anderson Cancer Center, Houston, TX; ^5^ Department of Head and Neck Surgery, The University of Texas MD Anderson Cancer Center, Houston, TX; ^6^ Cancer Diseases Hospital, Lusaka, Zambia

## Abstract

**PURPOSE:**

Formal education in the radiation sciences is critical for the safe and effective delivery of radiotherapy. Practices and patterns of radiation sciences education and trainee performance in the radiation sciences are poorly described. This study assesses the current state of radiation sciences education in Africa and evaluates a high-yield, on-site educational program in radiation biology and radiation physics for oncology and radiation therapy trainees in Africa.

**METHODS:**

An anonymous survey was distributed to members of the African Organization for Research and Treatment in Cancer Training Interest Group to assess current attitudes and practices toward radiation sciences education. A 2-week, on-site educational course in radiation biology and radiation physics was conducted at the Cancer Diseases Hospital in Lusaka, Zambia. Pre- and postcourse assessments in both disciplines were administered to gauge the effectiveness of an intensive high-yield course in the radiation sciences.

**RESULTS:**

Significant deficiencies were identified in radiation sciences education, especially in radiation biology. Lack of expert instructors in radiation biology was reported by half of all respondents and was the major contributing factor to deficient education in the radiation sciences. The educational course resulted in marked improvements in radiation biology assessment scores (median pre- and posttest scores, 27% and 55%, respectively; *P* < .0001) and radiation physics assessment scores (median pre- and posttest scores, 30% and 57.5%, respectively; *P* < .0001).

**CONCLUSION:**

Radiation sciences education in African oncology training programs is inadequate. International collaboration between expert radiation biology and radiation physics instructors can address this educational deficiency and improve trainee competence in the foundational radiation sciences that is critical for the safe and effective delivery of radiotherapy.

## INTRODUCTION

Radiotherapy is a critical component of definitive, adjuvant, and palliative treatment of many types of cancer. Because of its versatility, efficacy, and low overall cost compared with other treatments, radiotherapy remains one of the most widely used cancer treatments globally. The WHO estimates that there were 9.6 million cancer-related deaths globally in 2018, and that 70% of these deaths were in low- and middle-income countries (LMICs). Approximately 50% of patients with cancer globally will require radiotherapy during their disease course, but in LMICs, the International Atomic Energy Agency estimates that at least 60% of all patients with cancer would benefit from radiotherapy.^1-3^

**Context**

**Key Objective**
To understand current radiation sciences training practices in Africa and to pilot a high-yield course in radiation sciences for oncology trainees in Africa.
**Knowledge Generated**
Radiation sciences education for oncology trainees in Africa is inadequate. A high-yield, on-site course dramatically improves foundational and clinically relevant knowledge in radiation sciences.
**Relevance**
International collaboration can provide oncology trainees in Africa with foundational knowledge in the radiation sciences to ensure they are trained to deliver safe and effective radiotherapy.

Despite the tremendous need for radiotherapy in LMICs, access is poor because of limited availability of radiotherapy equipment and a severe shortage of oncologists.^4^ Africa is the least developed region in terms of radiotherapy capacity, with fewer than one external beam radiotherapy machine per 1 million people across the continent, compared with nearly 15 machines per 1 million people in North America.^4^ Compounding the effect of poor radiotherapy infrastructure is the dire shortage of cancer physicians in Africa, with several countries having no oncologist and many others having extremely high patient-to-oncologist ratios.^5^ A major limitation to expanding the shortage of African oncologists stems from the scarcity of clinical and/or radiation oncology training programs. Indeed, many practicing African oncologists were required to seek specialty training outside of their home country.^6^

Proficiency in the radiation sciences (ie, radiation biology and radiation physics) is required to provide safe and effective radiotherapy. Most oncology training programs incorporate formal instruction in the radiation sciences into training programs for radiation and clinical oncologists, and certifying bodies often require that oncologists prescribing radiotherapy pass one or more proficiency examinations in these subjects. The aim of this study was to survey and describe the current state of radiation sciences education in Africa and to evaluate the utility and effectiveness of a rigorous, 2-week, on-site course in radiation biology and radiation physics for clinical oncology and radiation therapy trainees from multiple African countries.

## METHODS

### Survey of Radiation Sciences Education

In January 2020, an online survey was sent to various specialists in radiation medicine in Africa who were members of the African Organization for Research and Treatment in Cancer (AORTIC). The survey assessed respondents’ country and institution; role; availability and type of radiotherapy treatments offered; the number and scope of practicing oncologists, medical physicists, dosimetrists, and radiation therapists; whether and how many oncology or radiotherapy (physics, dosimetry, therapy) trainees were engaged in formal training programs; and the current state and level of satisfaction with radiation sciences education programs for trainees. All responses were anonymous, and data were stored on the secure REDCap server at The University of Kansas School of Medicine.

### On-Site Educational Program in Radiation Biology and Radiation Physics

In June 2019, a group of practicing radiation oncologists and biologists (S.R.S. and C.M.T.) and radiation physicists (A.D.M. and J.D.O.) from The University of Texas MD Anderson Cancer Center traveled to the Cancer Diseases Hospital (CDH) in Lusaka, Zambia, to conduct an intensive, 2-week, on-site educational course in radiation biology (5 days) and radiation physics (5 days). Oncology and radiation therapy trainees from CDH and oncology trainees from other institutions in Tanzania, Kenya, Ghana, Rwanda, Lesotho, and Papua New Guinea attended the course on-site at the CDH. Lectures and interactive exercises were designed to cover the principles of radiation biology and radiation physics that are tested on the American Board of Radiology certifying examination for radiation oncology trainees in the United States. Radiation biology lecture topics were as follows: Radiation Matter Interactions; DNA Damage and DNA Repair; Cell Cycle and Cell/Tissue Kinetics; Mechanisms of Cell Death and Cell Survival Models; Time, Dose, and Fractionation; Oxygen Effects, Relative Biological Effectiveness, and Linear Energy Transfer; Molecular Signaling; Normal Tissue Responses, Acute Radiation Syndromes, and Radioprotection; Systemic Therapy, Radiation Modifying Drugs, and Hyperthermia; and Immune Modulation and Radiation Therapy. Radiation physics lecture topics were Atomic Structure and Radioactive Decay; X-Ray Creation and Delivery; Interaction of Photons with Matter and Measurement of Ionizing Radiation; Quantification, Measurement, and Calibration of Dose; Dose Distribution and Scatter Analysis; Monitor Unit Calculations; Dosimetry of Photon Beams in Homogenous Water Phantom; Electron Beam Therapy; Low-Dose-Rate Brachytherapy; High-Dose-Rate Brachytherapy; Prostate Implants and Accelerated Partial Breast Brachytherapy; Radiation Protection; Quality Assurance; and 3D-Conformal Radiotherapy and Intensity Modulated Radiotherapy.

For each discipline, a pretest examination was administered before the first lecture and an identical posttest examination was administered after the last lecture. The questions in the pre- and posttests are provided in the Data Supplement. The pretest also collected information related to the training program (oncology *v* radiation therapy) and a four-point scale (ie, very weak, weak, neither, and strong) of self-assessed knowledge in radiation biology and radiation physics. The posttest also included a trainee survey of the usefulness of each lecture, an assessment of the instructors, and an inquiry as to whether trainees felt better prepared to care for patients after taking the course. Pretest and posttest examination scores were compared between groups (oncology *v* radiation therapy) using the nonparametric Mann-Whitney test and two-way analysis of variance, and each trainee’s pre- and posttest performance was compared using the paired nonparametric Wilcoxon signed-rank test.

## RESULTS

### Survey of Radiation Sciences Education

**FIG 1:**
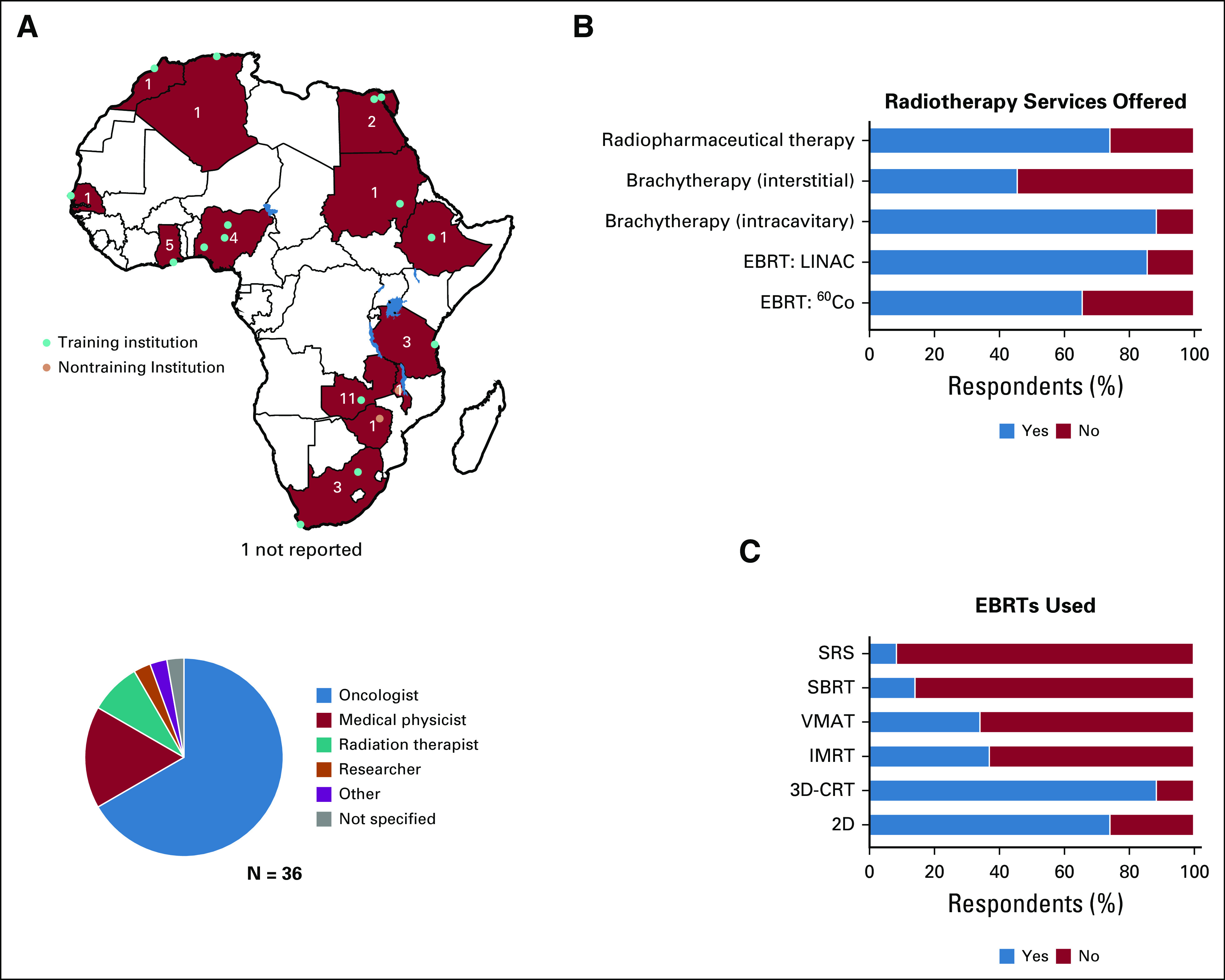
(A) Continental map depicting African Organization for Research and Treatment in Cancer Training Interest Group survey responses. Red countries denote at least one survey response was received, and inset numbers denote the total number of responses. Blue circles represent responses from training institutions, and pink circles denote responses from nontraining institutions. The pie chart below the map depicts the distribution of respondents’ roles. (B) Radiotherapy techniques used by respondents. (C) External beam radiotherapy techniques (EBRTs) used by respondents. 2D, two dimensional; 3D-CRT, three-dimensional conformal radiotherapy; ^60^Co, cobalt-60; EBRT, external beam radiotherapy; IMRT, intensity modulated radiotherapy; LINAC, linear accelerator; SBRT, stereotactic body radiotherapy; SRS, stereotactic radiosurgery; VMAT, volumetric modulated arc therapy.

Between January 9, 2020, and February 2, 2020, we received 36 responses from AORTIC Training Interest Group members practicing at 17 institutions in 13 African countries (Fig 1A). To gauge the spectrum of radiotherapy services provided and the educational needs of radiotherapy trainees (especially related to radiation physics), our survey assessed radiotherapy infrastructure and techniques used. Most respondents (67%) were practicing oncologists. More than 80% of respondents reported offering linear accelerator–based external beam radiotherapy (EBRT) and intracavitary brachytherapy, though a substantial percentage of respondents also offered radiopharmaceutical therapy, cobalt-60 teletherapy, and interstitial brachytherapy (Fig 1B). Among EBRT techniques used, three-dimensional conformal radiotherapy and two-dimensional (film-based) planning were the most common, though approximately 40% of respondents noted the availability of intensity modulated radiotherapy and approximately 33% offered volumetric modulated arc therapy. Use of stereotactic body radiotherapy. and stereotactic radiosurgery was only available in Egypt, Morocco, and South Africa.

**FIG 2:**
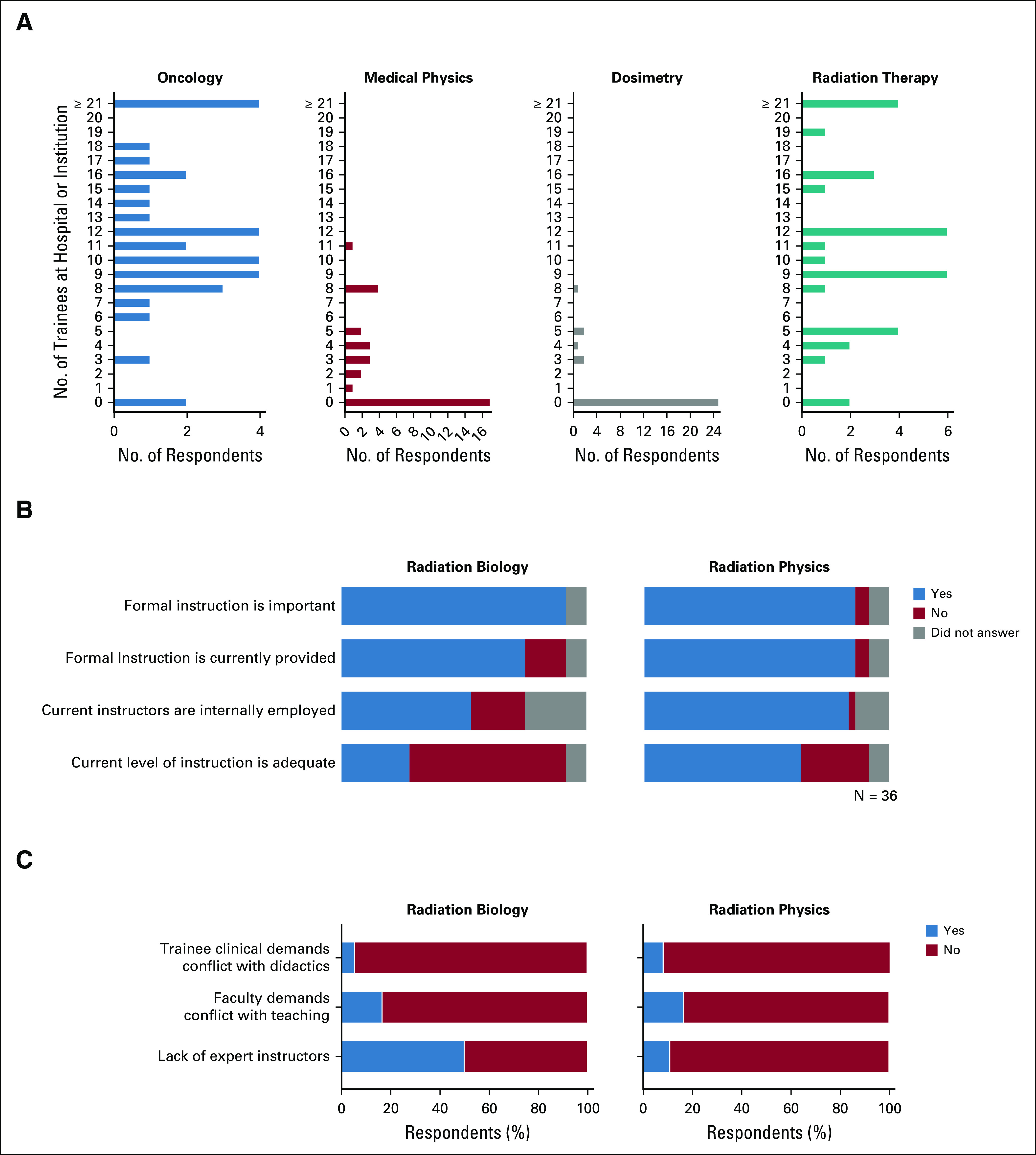
(A) Number and distribution of oncology and radiation therapy (medical physics, dosimetry, and therapy) trainees reported by survey respondents. (B) Current practices and attitudes toward radiation sciences training. (C) Challenges to effective instruction of radiation sciences.

Thirty-three of 36 respondents and 15 of 17 institutions reported having oncology or radiotherapy trainees enrolled in training programs at their institutions (Figs 1A and 2A). Among institutions with oncology trainees, 100% of respondents reported their programs provided training in radiotherapy, and the majority (96.7%) also provided training in chemotherapy, reflecting the common use of the clinical oncologist model in Africa. When surveyed about the current patterns of trainee instruction in radiation biology and radiation physics, most respondents felt formal instruction in these subjects is important, and most respondents reported that formal instruction is currently provided, most commonly by internal faculty or staff. Though most respondents reported some level of current training in both subjects, many felt the current level of formal instruction was inadequate, with 63.9% of respondents reporting inadequate instruction in radiation biology and 27.8% reporting inadequate instruction in radiation physics (Fig 2B). Various challenges were identified that prevent effective instruction, but the most notable and recurring theme was the lack of expert instructors in radiation biology (Fig 2C).

### On-Site Educational Program in Radiation Biology and Radiation Physics

**FIG 3:**
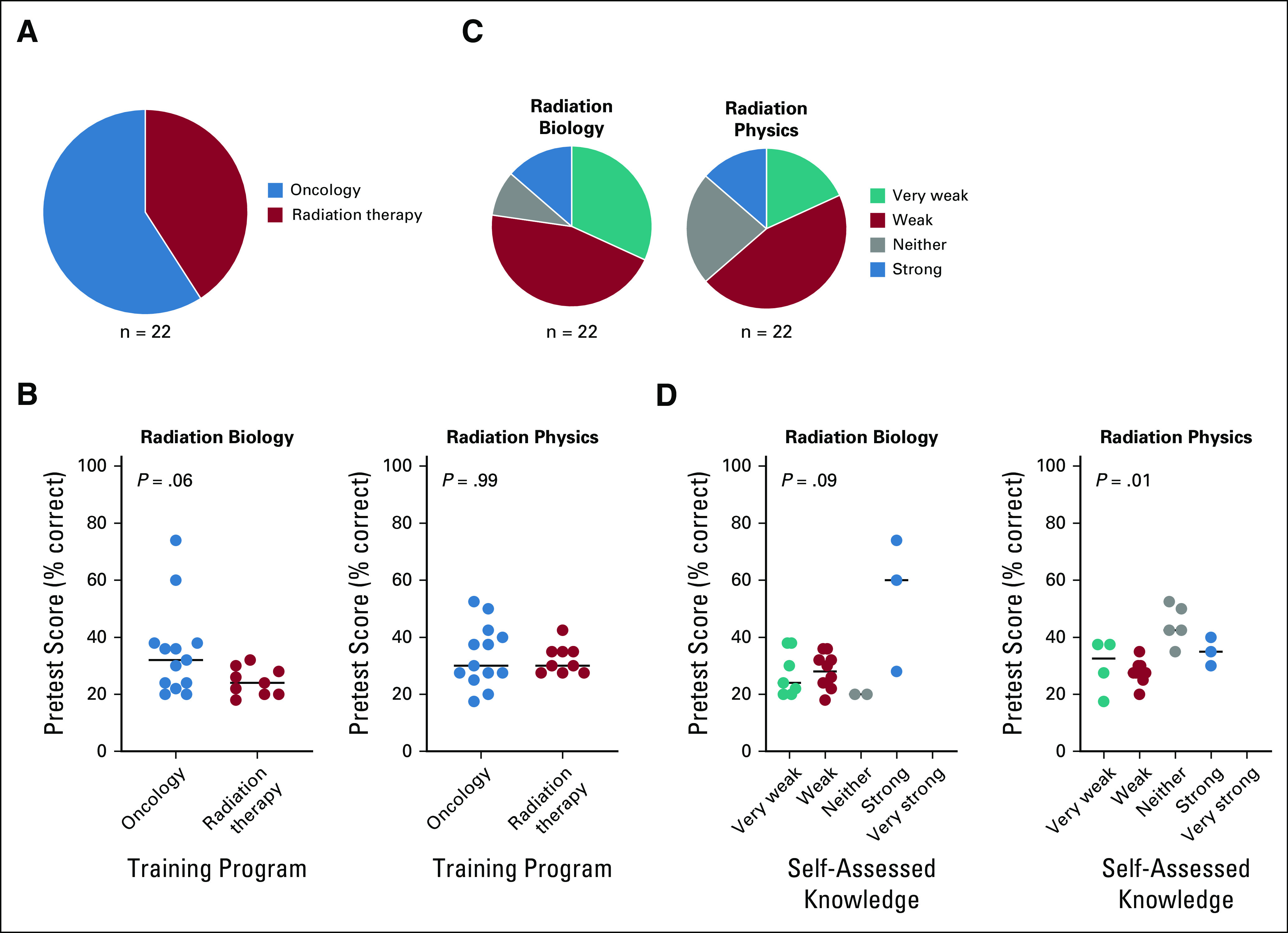
(A) Training program enrollment among trainees who attended the on-site radiation biology and radiation physics course at Cancer Diseases Hospital, Lusaka, Zambia. (B) Pretest scores in radiation biology and radiation physics by training program. Statistical test used was Mann-Whitney nonparametric *t* test. (C) Trainees’ self-assessed level of knowledge in radiation biology and radiation physics at the time of the pretest. (D) Pretest scores in radiation biology and radiation physics by self-assessed knowledge. Assessment was by two-way analysis of variance.

**FIG 4:**
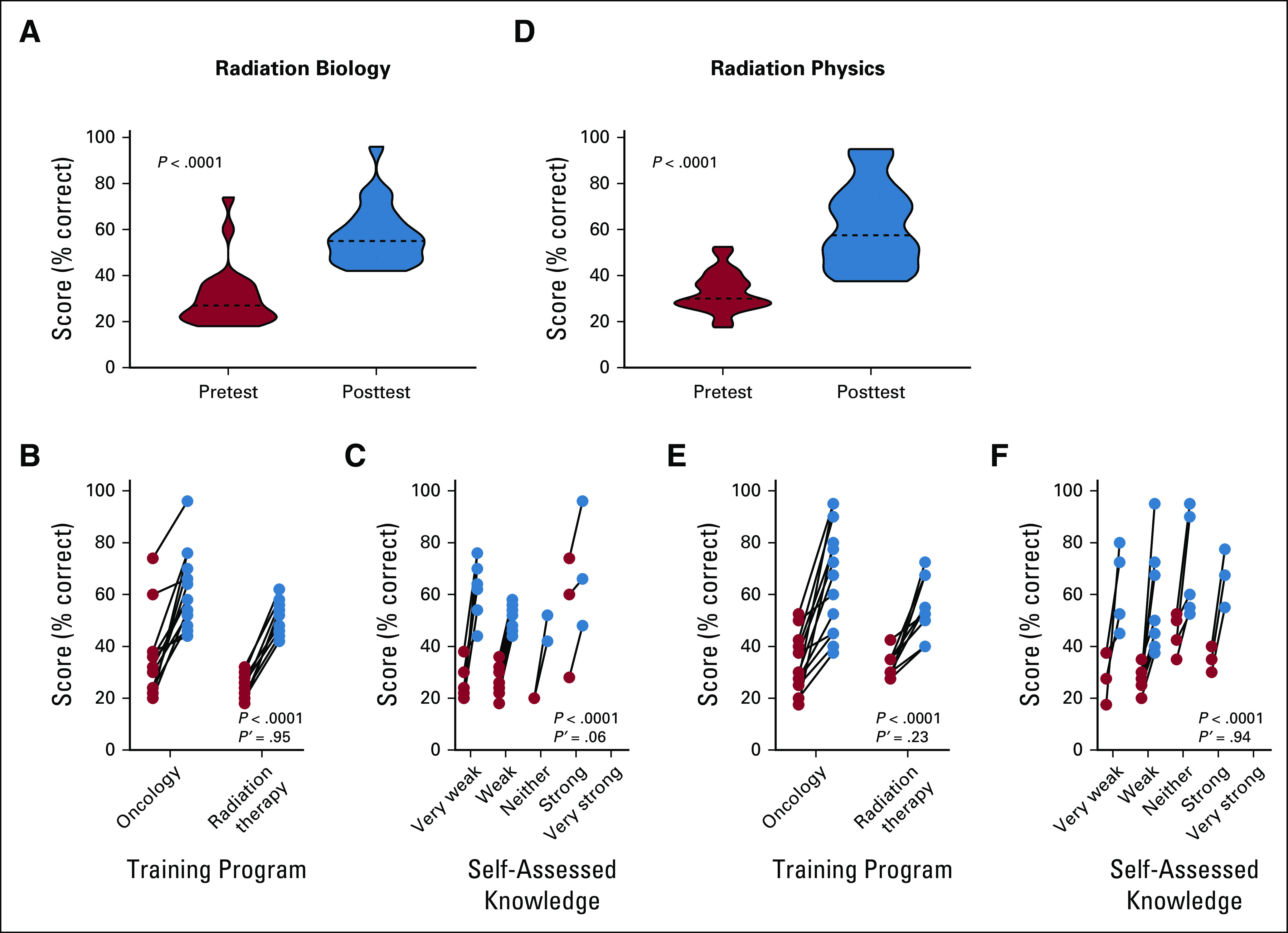
(A) Pre- and posttest scores in radiation biology for all trainees. Analysis was by paired nonparametric Wilcoxon signed-rank test. (B, C) Pre- and posttest scores in radiation biology based on (B) training program and (C) pretest self-assessed knowledge. Assessment was by two-way analysis of variance (ANOVA). *P* represents the comparison between pre- and posttest, *P*′ represents the interaction between (B) training program or (C) self-assessed knowledge and time (pre- *v* posttest). (D) Pre- and posttest scores in radiation physics for all trainees. Statistical test used was paired nonparametric Wilcoxon signed-rank test. (E, F) Pre- and posttest scores in radiation physics based on (E) training program and (F) pretest self-assessed knowledge. Assessment was by two-way ANOVA. *P* represents comparison between pre- and posttest, *P′* represents the interaction between (E) training program or (F) self-assessed knowledge and time (pre- *v* posttest).

We hypothesized that an on-site intensive course given by expert instructors in radiation biology and radiation physics would significantly enhance oncology and radiation therapy trainees’ knowledge base in these disciplines and that this enhanced knowledge would be reflected by improved performance on standardized examinations that assess core principles within the radiation sciences. Twenty-two trainees (n = 13 in oncology; n = 9 in radiation therapy) attended the 2-week on-site course at CDH (Fig 3A). There was a trend toward improved baseline knowledge in radiation biology among oncology trainees compared with radiation therapy trainees (*P* = .06) but no significant difference in baseline radiation physics knowledge (*P* = .99; Fig 3B). The distribution of baseline self-assessed knowledge in radiation biology and radiation physics among all trainees is shown in Figure 3C. Posttest assessment after the training course showed a marked and highly significant improvement in both radiation biology and radiation physics knowledge. For radiation biology, median pre- and posttest scores improved from 27% to 55%, respectively (*P* < .0001; Fig 4A). Improvements were highly significant for both oncology and radiation therapy trainees (*P* < .0001) and the magnitude of improvement did not differ based on training program (*P*′ = .95; Fig 4B). Similarly, improvement was seen across all self-assessed baseline knowledge groups (*P* < .0001), and there was trend toward interaction between self-assessed baseline knowledge and improved performance on the posttest examination (*P*′ = .06; Fig 4C). Improvements were similarly dramatic for radiation physics. Median pre- and posttest scores improved from 30% to 57.5%, respectively (*P* < .0001; Fig 4D). Improvements were highly significant for oncology trainees and for radiation therapy trainees (*P* < .0001) and the magnitude of improvement did not differ by training program (*P*′= .23; Fig 4E). Improvement was also seen across all self-assessed baseline knowledge groups (*P* < .0001), and there was no interaction between self-assessed baseline knowledge and improved performance on the posttest examination (*P*′ = .94; Fig 4F).

### Course Assessment

Trainees responded favorably to the course content and instructors. All trainees agreed or strongly agreed that the instructors were knowledgeable in the subject areas, that the lectures stimulated their interest in radiation biology and radiation physics, and that it is important that they understand the material to adequately care for patients. Every trainee responded affirmatively to the statement “I feel better prepared to care for patients because of this course.”

## DISCUSSION

Poor availability of radiotherapy infrastructure and a severe shortage of oncologists and radiotherapy personnel represent significant barriers to expanding access to life-saving radiotherapy in Africa. To address the human resources aspect of the cancer burden in Africa, it will be necessary to initiate training programs to dramatically increase the number and distribution of oncologists across the continent. Training programs in radiotherapy must ensure that oncologists possess a fundamental degree of knowledge in radiation biology and radiation physics, and formal instruction in these disciplines should be a component of all radiotherapy and clinical oncology training programs. This study identifies inadequacies in formal instruction in the radiation sciences, especially radiation biology, as significant and likely underappreciated problems within African oncology training programs.

A lack of expert instructors in radiation biology was identified as a primary obstacle to effective instruction. The percentage of radiation biology educators whose primary graduate work was in radiation biology has declined significantly over time.^7^ Indeed, even at many academic centers in developed nations, radiation biology is often taught by faculty whose graduate degree(s) lie outside of radiation biology. Individuals whose background is in cancer biology, molecular and cellular biology, or other related disciplines may have the requisite expertise to teach certain aspects of molecular radiation biology, but few educators are familiar with classical radiation biology, especially the theoretical and quantitative aspects of the discipline. The stewards of this knowledge are the few active, classically trained radiation biologists and the younger scientists and physicians whose research remains deeply immersed within the radiation sciences. In LMICs with even more limited access to expert radiation biology instructors, addressing this educational need for oncology trainees will likely require outreach efforts from international collaborators. The on-site educational course we conducted at CDH demonstrates that relatively short, intensive courses in radiation biology and radiation physics can dramatically improve performance in these disciplines and help trainees feel better prepared to care for patients. One limitation of our analysis is that, for logistical and data reliability concerns, we were unable to test retention of knowledge in the weeks to months after completing the course.

We currently host an annual on-site course in radiation biology and radiation physics at CDH that is open to trainees from other institutions, as well as ongoing teleconference educational sessions through the Project ECHO (Extension for Community Healthcare Outcomes) program for trainees at CDH. This approach allows us to provide continuity in educational outreach and telementoring to physicians and trainees in low-resource settings.^8^ It is our hope to expand our radiation sciences education program to include more trainees in LMICs, though these efforts will require ongoing commitment from multiple academic institutions and radiation sciences educators as well as support and investment from global stakeholders.
